# Supplementary material for: Are carbon emissions trading and green financial instruments synergistic? -Comprehensive quantitative research based on content analysis
Source: PLoS One. 2024 Mar 7;19(3):e0298601. doi: 10.1371/journal.pone.0298601 (PMC10919601; doi:10.1371/journal.pone.0298601)
Supplement: S1 Data — (ZIP) [file pone.0298601.s001.zip › Data/地级市Z_F工作报告.zip!/╔╜╢1⁄2/╫═▓⌐╩╨/└┤╫╘╣1⁄2╓┌║┼í╛┬φ┐╦╩2╛▌═°í┐_2016.docx]

各位代表：

现在，我代表市人民政府，向大会作政府工作报告，请予审议，并请各位政协委员和其他列席会议的同志提出意见。

一、“十二五”时期经济社会发展回顾

“十二五”时期是我市发展历史上极不平凡的五年。在中共淄博市委的坚强领导下，全市上下深入贯彻党的十八大和十八届三中、四中、五中全会及习近平总书记系列重要讲话精神，攻坚克难，砥砺奋进，全力做好稳增长、促改革、调结构、惠民生、防风险各项工作，扎实推动老工业城市转型升级，圆满完成“十二五”规划确定的各项目标任务，谱写了淄博科学发展的新篇章。

五年间，我们集中抓好经济社会发展的重大领域和关键环节，全市经济综合实力迈上了一个新台阶。2015年，预计全市地区生产总值实现4100亿元，是2010年的1.56倍；一般公共预算收入达到317.9亿元，是2010年的1.96倍；固定资产投资、社会消费品零售总额以及外贸出口分别达到2730亿元、1948亿元和57.9亿美元，分别是2010年的2.11倍、1.94倍和1.44倍；金融机构存、贷款余额达到3771.3亿元和2629亿元，分别是2010年的1.53倍和1.56倍；城镇和农村居民人均可支配收入分别达到33790元和14530元，分别是2010年的1.66倍和1.84倍。全市三次产业占比由2010年的3.7：61.6：34.7调整为3.5：54.2：42.3，呈现出结构不断优化、质量不断提升、协调性逐步增强的良好态势。

五年间，我们努力克服各种困难，办成了一批关系淄博长远发展的大事，区域发展水平显著提升。我市先后蝉联全国文明城市、国家卫生城市和全国“双拥模范城”，成功创建为国家森林城市和国家知识产权示范城市，跻身全国国土资源节约集约模范市和全国社会治安综合治理优秀市行列，并被确定为国家生态文明先行示范区、国家扶贫改革试验区和全国战略性新兴产业区域集聚发展试点城市，城市竞争力和影响力不断增强。

刚刚过去的2015年是“十二五”的收官之年。一年来，我们按照市委“一个定位、三个着力”的总体要求，积极应对经济下行的严峻考验，集中力量在加快结构调整、培育发展动能上求突破，在优化产业布局、推进集约发展上求突破，在加严环境保护、改善生态质量上求突破，在强化统筹协调、保障改善民生上求突破，在创新体制机制、增强发展活力上求突破，保持了经济社会平稳健康发展。全市地区生产总值和一般公共预算收入分别比上年增长7%和8.67%，固定资产投资、社会消费品零售总额和外贸出口分别增长14%、10.5%和3.4%（海关口径），城镇和农村居民人均可支配收入分别增长7.6%和8.7%，为“十二五”发展划上了圆满的句号，为“十三五”增创新优势、实现新跨越奠定了坚实基础。

一是转型升级取得积极进展。凝聚建设工业强市的强大共识，研究出台“工业强市30条”、“园区发展20条”等重要政策措施，全面启动淄博经济开发区、齐鲁化工区“一区四园”、主城区南部区域等重大生产力布局，市级财政兑现支持工业发展资金11.2亿元。坚持把优质项目建设和招商引资作为稳增长、强后劲的重要抓手，与8家省级银行签订授信金额1546亿元的战略合作协议，190个市重大项目完成投资420亿元，实际到位外来投资突破500亿元，中船重工、齐长城文化旅游创意园、汉能光伏、远成物流等一批重大招商项目扎实推进。深入实施创新驱动战略，国家重点实验室建设实现零的突破，高新技术产业产值占比达到31.7%（新口径）。现代物流、金融保险、电子商务、工业设计、软件信息等新兴业态快速发展，服务业占比提高1.6个百分点。积极应对严重干旱，粮食生产实现“十三连丰”，现代农业发展水平不断提高。

二是生态淄博建设加力提速。全面打响空气异味综合整治和孝妇河流域综合治理两场攻坚战，集中实施1237项环保治理工程和九大环保专项行动，全市“蓝繁”天数同比增加30天，空气质量良好率同比提高6.3个百分点，主要河流断面COD和氨氮平均浓度同比分别改善0.6%和25.7%，比“十一五”末分别改善30.4%和68.3%。开展火电企业超低排放改造和燃煤小锅炉集中整治，全面推广集中制粉、集中制气等清洁生产技术，成为全省高效煤粉锅炉试点示范市。大力推广型煤和兰炭，散煤无序排放问题得到破解。完成68处石灰石矿山关闭任务。大力开展美丽乡村连片创建示范行动，深入推进城乡环境综合整治和路域环境治理，加快农村危房改造和旱厕改造，实现了城乡环卫一体化全覆盖。

三是新型城镇化有序推进。淄博新区金融中心、文化中心、景观水系等功能项目进展顺利，齐盛湖公园、市图书馆新馆建成启用。济青高铁淄博段以及济青高速、滨莱高速淄博段扩容有序展开，联通路东延、东四路北延等一批城市路网工程建成通车，总投资10.44亿元的41个交通改造项目顺利实施，代表山东省圆满完成“迎国检”任务。开工建设农村住房1.35万户，新改建农村公路288公里，新解决16.1万农村人口饮水安全问题。

四是民生和社会建设全面提升。以提高群众满意度为着力点，民生支出占财政总支出的比重达到75.43%。全年新增城镇就业和农村劳动力转移就业8.9万人和7.4万人，城镇登记失业率2.81%。进一步提高各项社会保险待遇水平，降低冬季居民供暖价格，顺利完成保障性住房建设和棚户区改造任务。全年6万市定标准贫困人口实现脱贫，“十万农户脱贫奔康工程”如期完成。淄博三中新校、重庆路小学、市实验幼儿园等重点工程进展顺利，基本实现农村义务教育学校校舍标准化。县级公立医院综合改革取得新成果，基层医疗服务能力和公共卫生水平明显提升。启动文化名城建设，公共文化服务体系进一步完善，文化产业增加值占GDP比重达到3.6%。在第一届全国青年运动会上取得优异成绩。低生育水平保持稳定。国防动员和民兵预备役工作扎实推进。深化提升社会治理“三四二”工作格局，深入开展安全隐患排查整治集中行动，社会大局保持和谐稳定。统计、物价、广播电视、民族宗教、外事侨务、对台、防震减灾、气象、人防、史志、档案、关心下一代、妇女儿童、老龄、慈善、残疾人、红十字、援藏等各项事业都取得了新成绩、实现了新发展。

五是区域发展活力日益增强。强力推进“三最城市”建设，市级行政审批事项数量、审批时限和收费标准达到全省最低。在全省率先实施“三证合一”、“一照一码”等商事制度改革，全年新增市场主体7.5万户，同比增长149%。持续推进农村产权制度、财税体制、投融资体制等重点领域改革，出台企业资产顺位抵押政策，建立8支市级产业发展引导基金，齐鲁股权交易中心挂牌企业达到611家，25家企业在新三板挂牌。博山区、周村区重大区域政策争取以及重点地炼企业申请进口原油指标取得重要成果，陶博会和新材料技术论坛品牌影响力显著提升。

六是政府自身建设进一步加强。巩固党的群众路线教育实践活动成果，深入开展“三严三实”专题教育，建成全市统一的民生热线服务平台，完善“市长市民面对面”等制度安排，广泛倾听群众呼声，切实改进政府工作。积极推进法治政府建设，健全市政府重大决策专家咨询和法律顾问制度，公布市级行政权力清单，开通淄博市行政执法网，搭建市公共资源交易平台。自觉接受人大及其常委会的法律监督和政协的民主监督，虚心听取各民主党派、工商联和社会各界的意见，认真办理人大代表建议和政协委员提案。全面贯彻中央八项规定和省市委关于改进工作作风的一系列部署要求，严格落实党风廉政建设责任制，政府公信力、执行力不断提升。

各位代表！过去的五年，在中共淄博市委的坚强领导下，全市上下团结一心，开拓进取，谱写了在困境中奋进、在转型中发展的壮丽篇章。这五年，是老工业城市加速转型发展的五年，是淄博新的竞争优势不断增强的五年，是人民群众得到更多实惠的五年，是我们在加快建设全面小康社会道路上奋力前进的五年。五年的成就，得益于党中央、国务院和省委、省政府的科学决策和亲切关怀，得益于市委的正确领导，得益于市人大、市政协与社会各界的有力监督和大力支持，得益于全市广大干部群众负重奋斗、不懈努力。在此，我代表市人民政府，向全市广大干部群众，向各民主党派、工商联、各人民团体和社会各界人士，向中央、省驻淄单位、驻淄部队和武警官兵、政法干警，向海内外所有关心支持淄博改革发展的同志们、朋友们，表示衷心的感谢并致以诚挚的敬意！

在充分肯定成绩的同时，我们也清醒地看到，我市经济社会发展还面临一系列困难和问题，主要是：经济延续低位运行态势，稳增长的压力持续加大；结构性矛盾突出，支撑和带动转型升级的优质大项目不足，新的发展动力尚未形成；资源环境约束增强，生态环境脆弱，治理和改善生态环境特别是大气质量任务艰巨；社会治理面临许多新课题，安全生产、市场出清、民生改善等方面矛盾易发多发；各项改革有待进一步深化，政府自身建设任重道远，等等。对这些困难和问题，我们必须高度重视，采取更加扎实有效的措施，全力认真解决。

二、“十三五”时期经济社会发展的总体思路和目标任务

“十三五”规划是经济发展进入新常态后第一个五年规划。根据党的十八大和十八届三中、四中、五中全会精神以及市委十一届十一次全会精神，市政府编制了《淄博市国民经济和社会发展第十三个五年规划纲要（草案）》。《纲要（草案）》及《说明》已印发大会，请一并予以审议。

“十三五”时期是全面建成小康社会、实现第一个百年奋斗目标的决胜阶段，也是加快推进淄博转型发展的决战时期。今后五年，我们要高举中国特色社会主义伟大旗帜，以邓小平理论、“三个代表”重要思想、科学发展观为指导，深入贯彻落实党的十八大和十八届三中、四中、五中全会及习近平总书记系列重要讲话精神，紧紧围绕“四个全面”战略布局，坚持发展第一要务，以提高发展质量和效益为中心，全面践行创新、协调、绿色、开放、共享发展理念，以走在前列为目标定位，着力建设工业强市、文化名城、生态淄博，提前全面建成小康社会，开创淄博科学发展新局面。为此：

《规划纲要（草案）》全面贯彻了创新、协调、绿色、开放、共享的发展理念，将其作为破解发展难题，增强发展动力，厚植发展优势，实现提前全面建成小康社会奋斗目标的根本指导和遵循，自觉贯穿于转型发展的全过程。

《规划纲要（草案）》集中体现了市委“一个定位、三个着力”总体要求和实现“十个新突破”的目标任务，紧紧抓住工业强市、文化名城、生态淄博建设的重点领域和关键环节，集中用力、持续加力，以坚定的信念和决胜的力度，推动全市各项事业走在前列、实现跨越。

《规划纲要（草案）》突出强化了推动经济转型和城市转型两大战略任务，集中在培育发展新动力、拓展发展新空间、构建产业新体系上精准发力，推动发展方式从要素驱动向创新驱动转变，产业结构从中低端向中高端转变，城市形态由传统城市向现代城市转变。

《规划纲要（草案）》充分考虑了我市未来发展趋势、发展条件和提前实现“两个翻番”的发展目标，提出了“十三五”时期经济社会主要发展目标及重点任务，包括八个方面：

一是经济综合实力显著提升。在提高发展平衡性、包容性、可持续性的基础上，全市地区生产总值和居民人均可支配收入分别于2019年和2018年实现比2010年翻一番。到2020年，全市地区生产总值突破5600亿元，年均增长7.5%以上；居民人均可支配收入突破4.1万元，一般公共预算收入突破480亿元，年均分别增长8.5%以上；固定资产投资和社会消费品零售总额年均分别增长15%和12%左右。

二是产业结构层次显著提升。重点实施创新型企业培育工程、创新平台建设工程、关键共性技术攻关工程，建设鲁中创业创新中心，推动淄博高新区与中关村共建产业基地，打造山东半岛国家自主创新示范区（淄博园）。到2020年，全市规模以上企业研发经费投入占企业主营业务收入比重达到1.2％以上，全社会研究与试验发展经费支出占GDP比重达到2.7％。以高端化、绿色化、智能化为导向，加快化工、机械、建材、冶金、纺织等五大主导产业改造提升，实现新材料、生物医药、机电装备制造、新能源及节能环保、电子信息等五大新兴产业倍增发展，做大做强现代物流、金融保险、信息服务、工业设计、商务咨询等五大生产性服务业，基本建立以先进制造业和现代服务业为主导的现代产业体系。推进农业生产标准化、规模化、产业化，打造国家现代农业示范区。到2020年，规模以上工业增加值率提高到22%以上，高新技术产业产值占比达到36%以上，服务业增加值占地区生产总值比重达到50%左右。

三是文化软实力显著提升。打造齐文化传承创新示范区和国内知名文化旅游目的地城市，培育壮大文化旅游、创意会展、影视（动漫）传媒、印刷出版、工艺美术五大优势文化产业。到2020年，全市文化产业增加值占GDP比重达到4.5%以上。实施文化惠民工程，构建15—20分钟城乡公共文化服务圈。市民素质和社会文明程度显著提高，蝉联全国文明城市称号。

四是生态环境质量显著提升。以国家生态文明先行示范区建设为载体，持续推进大气、水和土壤污染防治，推动绿色循环低碳发展。到2020年，四项空气主要污染物浓度累计下降30%以上，环境空气质量优良天数占比力争达到70%，全市70%以上的流域水系达到Ⅲ类水体。强化生态保护和生态修复，构建主城区“生态屏障”。到2020年,全市森林覆盖率和建成区绿化覆盖率分别达到38%和48%以上。

五是城乡统筹发展水平显著提升。突出主城区核心引领作用，推动中部淄川、博山、周村、临淄、桓台转型发展，打造北部高青健康服务业和南部沂源生态经济示范区。到2020年，全市常住人口和户籍人口城镇化率分别达到73%和63%以上，主城区实现全域城市化，城镇化水平继续走在全省前列。策划实施一批事关全市长远发展的重大基础设施项目，打造“三横一纵”客运铁路网、“三横三纵”货运铁路网、“三横两纵”高速公路网以及“六横四纵”区际干线路网;积极推进城市轨道交通建设，启动通用机场建设前期工作；全面建成“五交两直”特超高压为支撑的坚强智能电网；加快建设海绵城市、坚韧城市和智慧城市。

六是开放型经济水平显著提升。积极融入“一带一路”以及京津冀协同发展、全省“两区一圈一带”等区域发展战略，加快建设中德工业4.0示范区、中以淄博创新谷、区域股权交易市场、综合保税区、跨境电商平台、境外经贸合作园区、境外营销展示中心等开放合作载体，提升对外贸易质量和效益。“十三五”期间，全市累计利用外来投资突破2800亿元。到2020年，外贸进出口总额突破120亿美元。

七是居民生活水平和质量显著提升。积极推进国家扶贫改革试验区建设，确保今年率先实现贫困人口基本脱贫；加快实施城市棚户区改造、老旧居住区整治，农村社区改造和“五化”建设，全面提升城乡居住环境；到2020年，累计新增城镇就业35万人，城乡基本养老保险和医疗保险覆盖率分别达到92%和98%，高水平普及15年基础教育，人均拥有公共文化体育设施面积达到2平方米。

八是发展环境质量显著提升。全面完成中央和省市委全面深化改革任务目标，“三最城市”建设成果更加巩固，企业发展环境和政府服务环境明显改善，法治淄博、平安淄博、诚信淄博建设取得重大进展，全社会创业创新活力得到充分释放。到2020年，全市市场主体数量和社会融资总量分别突破50万户和800亿元。

各位代表！ “十三五”是我市转型发展的关键时期，有压力、有挑战，更有机遇，我们一定要主动顺应新常态下速度变化、结构优化、动力转换的发展趋势，牢牢把握“一个定位、三个着力”的总体要求，始终保持开拓进取、奋发有为的精神状态，勇于担当，积极作为，全力推动淄博转型发展爬坡过坎。我们坚信，有市委的坚强领导，有“十二五”发展打下的良好基础，有广大人民群众的信任和支持，只要我们凝神聚力、真抓实干，就一定能够战胜各种艰难险阻，把“十三五”的壮丽蓝图变为蓬勃发展的美好现实。

三、2016年经济社会发展的目标任务和工作重点

2016年是“十三五”的开局之年，是全面建成小康社会决胜阶段的第一年。根据市委关于今年经济社会发展的总体部署，今年全市经济社会发展的预期目标建议为：地区生产总值增长7.5%左右，一般公共预算收入增长7.5%左右，固定资产投资增长15%左右，社会消费品零售总额增长11%左右，进出口保持稳定，实际到账外资增长6%左右，城镇和农村居民人均可支配收入分别增长8%和8.5%左右，城镇登记失业率控制在4%以内，全面超额完成省下达的节能减排约束性目标。

为确保“十三五”良好开局，我们紧紧抓住优质项目建设这个转型发展的“牛鼻子”，自去年7月份开始，集中对2016年度重点项目进行策划论证，确定全市重大项目290个，总投资2478亿元，年度投资798亿元，其中投资10亿元以上的项目69个。化工产业项目大幅减少，新兴产业项目占到工业项目的58%，文化旅游、生产性服务业、生态环保和社会民生项目比上年增加1倍以上，也包括涉及年度投资58亿元的“绿动力提升工程”，34亿元的11个水系建设和生态修复提升项目，58亿元的棚户区改造和老旧居住区整治项目以及31亿元的21个教育、医疗项目。同时，还确定了年度投资247.8亿元的113个主城区及全市统筹城建重点项目。目前，这些项目已全部落实了市政府牵头负责人和具体责任单位，明确了融资渠道和要素保障部门，部分项目已展开前期工作。这是今后发展的潜力所在、后劲所在 ，我们有信心、有决心认真扎实地实施好这批大项目、好项目，有信心、有决心在新一轮产业升级中实现加速换挡、弯道超越。具体措施上，将按照“一个定位、三个着力”总体要求，紧紧围绕实现“十个新突破”，努力做到“一个确保、三个着力、四个加快”：

（一）确保经济实现中高速增长

一是全力稳定经济运行。全面落实中央、省出台的各项减税清费和降低企业运营成本的政策措施，推行行政事业性收费、政府性基金和涉企收费目录清单，清理规范行政审批中介服务，坚决兑现“工业强市30条”政策，增强企业抵御经济下行和在困境中谋发展的信心和能力。强化经济运行监测，建立完善覆盖各经济领域和重点工程项目、重大经济活动的调度、分析机制，有效应对经济形势变化。

二是切实加大有效投入。紧紧抓住290个年度重大项目，以及首批总投资195亿元的13个利用国家政策性贷款项目，细化落实“三个跟着走”的工作推进机制，全力以赴保开工、保建设、保质量、保达效。围绕转型发展的重点区域、关键环节，继续策划一批大项目、好项目，进一步加大对国家专项建设基金、重大区域政策以及政策性贷款的争取力度，适度新增地方政府债券规模，带动有效投资持续增长。

三是加强财税运行管理。发挥政府性资金的牵引功能，运作好并购重组、股权投资等专业化引导基金，积极推进济青高铁淄博段建设等社会资本合作项目，以政府购买融资服务模式推进棚户区改造和老旧居住区整治，吸引更多社会资本投向我市经济社会发展的薄弱环节和关键领域。加大税收征管力度，全面推进国地税联合办税，充分挖掘税源潜力，力争地方财政收入税收占比提高2个百分点以上。

四是大力强化金融保障。持续深化政银企合作，用力提高抵质押贷款比率，实施面向小微企业的普惠金融服务网格化试点工程，有效缓解企业融资难、融资贵问题。强化齐鲁股权交易中心辐射带动功能，支持上市企业再融资，力争年内境内外上市和新三板挂牌企业分别突破30家和40家。高度重视金融风险防控，进一步完善和提升主办行制度，扎实做好大额信用风险处置和复杂担保圈破解，高度关注并持续化解部分企业出现的风险问题；继续严厉打击恶意逃贷、金融诈骗、非法集资等违法犯罪行为，坚决守住不发生区域性金融风险的底线。

（二）着力推进工业强市建设

一是构建产业新体系。紧紧围绕绿色、智能、集约化发展，以技术改造、园区建设和优质大项目招商为主攻点，全力推进传统产业结构升级、新兴产业倍增发展。以工艺改进、装备升级和智能化改造为着力点，集中推进总投资300亿元左右的150个重点技改项目，对化工、机械、建材、纺织等传统产业进行数字化、网络化、智能化改造，形成新的比较优势。以新材料、生物医药、机电装备制造、新能源及节能环保、新一代信息技术等产业为重点，深入实施高新技术产业“铸链工程”，全力抓好中船重工动力电源、汉能薄膜太阳能电池、北汽新能源汽车、新华医疗生物制药装备等一批重大新兴产业项目，以及总投资44.6亿元的76项创新发展重点项目，培育具有高成长潜力的产业梯队，年内高新技术产业产值占比提高1个百分点以上。顺应服务业与制造业加快融合的趋势，扎实推进远成物流、传化金泰公路港、太保在线服务中心、卓创资讯信息服务平台等年度投资180亿元的126个服务业重点项目，全力打造鲁中物流中心、区域性金融中心、鲁中设计创意中心和互联网淄博产业带，年内服务业占比提高1.5个百分点以上。

二是发展壮大园区经济。市级重点抓好淄博高新区、淄博新区、淄博经济开发区、主城区南部区域、齐鲁化工区“一区四园”和文昌湖区等六大板块，各区县重点抓好省级经济开发区和产业聚集区。突出抓好高端规划、产业定位、低差企业退出、优质骨干企业培育、配套设施整合建设五项重点工作，下功夫在培育主导产业、完善基础设施、提升生态功能、促进集约发展上全力突破。用3年时间基本完成以主城区南部区域为主体的建陶、化工和物流产业布局调整，“十三五”期间，全市化工产业集中度达到80%以上，园区经济占全市工业经济的比重达到80%以上，努力把园区建设成为优势产业的聚集区、开放创新的先行区、绿色集约发展的示范区。

三是突出培植龙头企业。重点扶持“双50强”以及各行业具备领军素质的企业，带动形成一批技术领先、核心竞争力强、规模与品牌优势突出的企业集群。充分认识产业结构调整形成的企业出清机遇，紧紧抓住企业兼并重组这个重大机遇“窗口”，引导支持企业走资本运作、兼并重组的路子，尽可能地通过兼并重组吸附优势要素补齐发展短板。不断深化与中央、省驻淄企业的融合发展，全力支持齐鲁石化、中铝山东企业一批重大项目建设。大力实施品牌带动和标准化战略，深入推进规模以上企业规范化公司制改制“五年行动计划”，组织所有规模以上企业以数字化、网络化和智能化为重点，分行业开展企业管理对标，下决心改变粗放式管理模式。

四是积极化解过剩产能。要把化解过剩产能作为深化结构性改革的重大任务，对“低、重、差”企业和资不抵债、扭亏无望的僵尸企业，充分运用节能环保、安全生产、技术标准等倒逼机制，推动重组整合、搬迁改造或破产关闭，加快实现市场出清，为优势产业腾出要素资源和发展空间。坚持多兼并重组重整、少破产清算，对确需破产关闭的企业，制定安全可行的关停方案和维稳措施，有效解决不良资产处置、失业人员再就业和生活保障等关键问题，努力把化解过剩产能带来的社会成本降到最低。

（三）着力推进文化名城建设

一是加大齐文化保护开发力度。组建齐文化研究院，办好稷下学宫论坛和齐文化节，深度挖掘齐文化开放、包容、创新、重商的精髓。推进齐国故城考古遗址公园、田齐王陵遗址公园、齐都文化城联动开发，组织实施齐国故城遗址、陈庄—唐口遗址等国家级重点文物保护工程，抓好“齐国故都与齐王陵”申报世界文化遗产工作，努力把资源优势转化为产业优势。

二是加速文化与产业融合发展。依托丰富的自然资源和文化遗存，加强高端策划，统筹抓好齐国故都文化游、齐长城文化旅游创意园、周村古商城三期、陶琉古镇、华侨城旅游综合体等重点项目，充分挖掘“中华老字号”和特色旅游产品潜力，集中打造国内知名旅游目的地。实施“乡村记忆”工程，推进博山池上、淄川梦泉等10个乡村旅游片区的开发提升，培育一批功能完备、服务规范的示范点。实施动漫产业突破工程，加快发展文化创意、游戏动漫、设计服务、博古文玩等新兴文化业态，继续办好陶博会和新材料技术论坛等品牌展会，努力把文化产业培育成为全市支柱产业。

三是加强公共文化服务体系建设。加快建设中国陶琉馆、市科技馆、群艺馆、美术馆、大剧院等新区文化中心重点工程，建设改造高新区文体中心等一批区县重点文化项目，进一步完善农村社区文化服务中心等镇村文化设施，加快打造15-20分钟城乡公共文化服务圈。

（四）着力推进生态淄博建设

一是坚决打好大气和水污染防治攻坚战。继续开展空气异味综合整治。下大气力抓好电力、钢铁、建材、焦化等行业的二氧化硫、氮氧化物和粉烟尘治理，建筑、交通、物流等行业的扬尘污染治理，石化、医药、家具等行业的挥发性有机物治理，对1064家重点企业逐一进行整治，确保稳定达标排放。深入推进孝妇河流域综合治理。新建改造文昌湖污水处理厂等4座城镇污水处理厂，实施张店污水处理厂搬迁改造，全面完成污染点源治理和湿地建设任务，确保流域水质稳定达到Ⅳ类水标准。大力实施“绿动力提升工程”。强力推进电站锅炉超低排放改造、园区集中煤制气、高效煤粉锅炉改造、陶瓷干法集中制粉、直燃煤小锅炉清洁能源置换等重点工作。组织开展窑炉专项治理。继续抓好散煤治理，力争型煤和兰炭用量提高到30%以上。今年全市空气良好天数要达到160天，“蓝繁”天数突破50%，空气质量指数实现全省位次前移，16个主要河流断面稳定达到消除“劣五类”标准。

二是进一步加大生态修复力度。突出抓好破损山体特别是四宝山、九顶山、山铝赤泥堆和采石矿区等重点区域生态修复提升，以及张店公园、青年公园、花山公园等园林绿地改造建设，新增造林面积10万亩、园林绿地面积300公顷。抓好南部山区水土保持重点项目，加强东猪龙河、涝淄河、乌河等重点流域治理，组织实施主城区“八河联通、六水共用、清水润城”工程，加快建设和提升孝妇河、五阳湖、马踏湖、文昌湖等湿地公园，努力打造“大水系”生态格局。深入开展土壤污染治理与修复，加大农业面源污染防治力度，用最严格的措施抓好饮用水水源地保护和危险废物处置管理，确保生态环境安全。

三是健全生态建设长效机制。加快划定全市生态红线，严格实行环境准入制度，加大生态补偿和转移支付力度，坚决守住生态安全底线。全面落实环境总量控制、能评、环评、排污许可等制度，坚决把严新建项目节能环保门槛。实施工业污染源全部达标排放计划，开展乡镇和工业园区空气质量监测，实现市控以上重点企业、重点区域在线监测全覆盖。进一步加强环境信息公开，建立网络举报平台，持续加大“刑责治污”力度，对环境违法犯罪行为严查重处、绝不姑息。

（五）加快实施创新驱动发展战略

一是大力强化科技创新支撑功能。实施创新能力提升工程，加快山东新材料产业技术研究院建设，推动省级重点实验室等各类研发机构晋档升级。积极拓展对外科技合作，充分发挥慕尼黑、硅谷等海外孵化器招引聚集作用，全力打造中德工业4.0示范区、中以淄博创新谷等国际创新合作平台，吸引汇聚更多海外高端创新人才、优质科技资源和先进技术成果。深入推进“两化”融合发展，支持龙头企业建设智能工厂，推动100家企业开展“机器换人”，着力打造省级“两化”融合示范区和智能制造产业集聚区。

二是大力推进“大众创业、万众创新”。重点建设齐鲁创业创新谷、山东理工大学创业创新学院等高品质创业创新平台，进一步完善以市大学生创业孵化中心和创业梦工场为龙头的创业孵化体系，为创客人才和初创企业提供全程服务。积极引进和组建各类天使、创业和产业投资基金，鼓励大企业开展定制化生产、众筹众包等商业模式创新。高度重视园区外绿色小企业集群的发展，以小而特、小而强为目标，培育一批品牌化、创新型“小巨人”。高标准推进国家知识产权示范城市建设，深化商事制度改革，充分激发全社会的创业创新热情。

三是大力加强人才队伍建设。实施“淄博英才计划”，加大对国内外高层次人才，特别是“长江学者”、“泰山学者”等领军人才的引进力度，为创新驱动提供强大智力支撑。深入实施企业家十年培训计划，加快打造一支具有国际视野和社会价值追求的高素质企业家队伍。健全完善股权、期权、分红等激励机制，大力弘扬鼓励创新、宽容失败的社会文化，为各类人才施展才能创造良好环境。

（六）加快推进城乡统筹发展

一是进一步优化城市空间布局。按照“中心凸显、十字展开、组团特色、融合发展”的总体思路，集中力量做优做强主城区。淄博新区要抓好文化中心、新建高中等五所中小学、市中心医院西院区等三大医疗项目以及天津路等一批重点公共设施，实施金融街、黄金电商产业园等一批高端服务业项目，切实增强综合承载功能；老城区要突出抓好棚户区改造和老旧居住区整治，以及农贸市场、背街小巷、路网管网建设改造工程，努力破解交通拥堵、管网不畅、管理缺失等“城市病”。加强主城区与次城区的互联互通，重点实施南部产业结构调整和马南路建设、西五路南延、世纪路南段（昌国路至华福大道）配套设施改造，北部西六路、世纪路济青高速跨线桥改造和高铁新城规划建设，东部金晶大道（人民路至洪沟路）、新村路（东四路至金晶大道）改造和人民路、华光路下穿淄东铁路桥立交通道工程，西部鲁泰大道和人民路西延、姜萌路南延以及柳园路北延等工程。高标准完善各组团间生态廊道，优化提升次城区的功能分区，构建梯次带动、优势互补、协同发展的城镇化格局。

二是切实增强城市承载辐射能力。积极推进济青高铁淄博段建设，全力支持济青高速、滨莱高速改扩建和长深高速建设，集中实施S102济青线淄博段改建等9个国省道建设改造项目。同时，积极争取沾临高速淄博段、济潍高速淄博段列入省“十三五”规划，并尽早展开前期规划论证工作。继续抓好南水北调地方配套和引黄供水配套工程，实施主城区供水系统改造及刘征水源地应急供水工程；扎实推进中石化济青二线淄博段、第二环保热电厂、“三防一青”综合教育训练基地等重点基础设施建设；加快实现以地理信息为基础的信息资源共享，建设智慧城市。实施城市精细化管理工程，突出全域管理、精细管理，对环境卫生、交通秩序、小区物业、户外广告、渣土清运、噪声扰民和违规建设等突出问题，列出清单，压实责任，彻底整治，让群众切实感受到居住环境的提升改善。

三是扎实推进“人”的城镇化。深化户籍制度改革，增强城市对农业转移人口的吸纳能力，力争今年常住人口和户籍人口城镇化率分别提高1.5个百分点以上。突出抓好全国重点镇、省市级示范镇建设，推动处在十字轴线上的小城镇整建制城镇化，带动农村人口就地市民化。深入实施美丽乡村连片创建，加快基础设施和公共服务设施向农村延伸，建设一批家园美、田园美、生态美、生活美的宜居村庄。注重把“人”的城镇化与化解房地产库存有机结合起来，鼓励进城务工人员在城镇购房，鼓励符合住房保障条件的家庭通过房产租赁市场承租住房。加大棚户区改造货币化安置力度，探索“房票”制度，大幅度提高货币化安置率。

四是加快发展现代农业。落实强农惠农政策，抓好粮食高产创建，加快发展特色农业，扩大“三品一标”优质农产品规模，打造农产品质量安全放心城市。实施“互联网+现代农业”行动，建立电子商务与实体流通相结合的营销体系，培育一批特色农产品“淘宝镇”、“淘宝村”。加强农村基础设施建设，突出抓好中小河流治理、雨洪资源利用、抗旱应急水源等项目，确保农村地区人畜饮水安全。继续深化农业农村改革，完善农村集体产权权能，发展多种形式适度规模经营。积极推动农产品生产、加工、销售与都市农业、乡村旅游、餐饮服务融合发展，带动农业增效和农民增收。

（七）加快提升民生和社会建设

一是坚决打赢脱贫攻坚战。年内实现市现行扶贫标准下7.5万贫困人口基本脱贫，是今年乃至“十三五”时期全市民生建设的头等大事。我们将全面贯彻市委决策部署，在搞好精准识别的基础上，坚持因地制宜、因人制宜，通过产业扶贫、就业创业、社保兜底等多种措施，坚决打赢脱贫攻坚战。

二是统筹做好城乡就业和社会保障工作。深入实施高校毕业生就业促进计划和农民工职业技能提升计划，全年培训城乡劳动者5万人，城镇新增就业7万人。加快推进“全民参保登记计划”，努力实现应保尽保。进一步完善临时救助、大病救助、慈善救助等制度。支持发展医养融合型养老机构和养老产业，努力打造功能完善、规模适度、覆盖城乡的养老服务体系。

三是提高教育、卫生等社会事业发展水平。推动县域义务教育均衡发展，博山区、沂源县通过国家义务教育发展基本均衡县认定。把解决“大班额”问题作为优化教育资源配置的硬任务，新开工建设26所中小学、改扩建36所中小学，2017年全面完成62所中小学的新改扩建，全面改善农村义务教育薄弱学校办学条件。继续推进公立医院综合改革，完善基本药物制度和分级诊疗制度，年内15%以上的乡镇卫生院达到甲级标准，基本公共卫生服务实现城乡居民全覆盖。加快发展健康服务业，鼓励社会办医。实施全面两孩政策，继续改善出生人口性别比，对放开两孩政策带来的教育、医疗等问题研究具体解决措施。大力开展全民健身运动，提高竞技体育水平，积极发展体育产业。

四是抓好重大民生实事。一要加快棚户区改造和保障性住房建设，年内改造城市棚户区2.44万户。二要加快城市老旧居住区整治，年内完成68个老旧居住区的整治任务，用4年时间，对全市1995年以前建设的老旧居住区全部整治一遍。三要加快便民农贸市场规划建设，年内新建、改建、提升一批便民市场，合理设置一批季节性摊点、临时疏导安置区及早夜市，实现规范管理。四要加快推进农村“五化”改造提升工程，集中支持224个省扶贫工作重点村实施“五化”建设，全面完成农村存量危房改造任务，对574个村、9.05万户实行旱厕改造。

五是加强和创新社会治理。以建设法治淄博为目标，深化社会治理“三四二”工作格局。把安全生产作为任何人、任何时候都不能碰触的“高压线”，彻底细化实化各级各部门安全监管责任清单和企业主体责任清单，完善安全监管物联网系统，大力开展隐患排查整治，积极推进化工产业布局调整，组织实施消防、铁矿采空区和液化气钢瓶专项整治行动，坚决杜绝重大事故发生。严格推行“刑责治安”，落实企业“黑名单”和安全生产“一票否决”制度，对安全生产违法行为和责任人依法从严惩处。积极开展食品安全示范创建工作，确保群众生命健康安全。健全社会治安立体防控体系，继续推进视频全覆盖工程，实现对社会治安要素的全面把控。落实信访工作责任制、领导干部公开接访、挂包解决疑难信访问题等制度，切实推进依法治访，提高信访事项办结率和群众满意度。

（八）加快推动改革开放

一是持续深化重点领域改革。继续深化投融资体制改革。对投资准入实行负面清单管理，减少和规范投资项目前置审批。组建运营好市属国有资本投资运营公司、市金融控股集团有限公司和市文化旅游资产经营有限责任公司，进一步提升融资带动能力。扎实深化财税金融改革。积极推进“营改增”试点扩面。加大财政预决算公开力度，今年起，除涉密部门外，所有使用财政资金的部门全部公开预决算。稳步实施新型农村合作金融试点。深化价格改革，落实阶梯水价政策，完善阶梯电价、气价政策。大力推行全市公交集约化改革，加快实现城乡公交一体化。全面推进不动产统一登记制度改革。

二是持续提高开放型经济水平。全力推动专业化精准招商，更多采取并购重组、引入战略投资等方式，鼓励我市企业与国内外大企业、大集团进行资本、品牌、技术和渠道对接，以优质增量带动激活存量，力争全年外来投资增长15％以上。全面落实国家、省稳定外贸增长的政策措施，加大骨干出口企业扶持力度，用好阿里巴巴“一达通”外贸综合服务平台，加快新华锦（淄博）跨境电子商务平台建设，更多依靠技术、品牌、质量、服务开拓国际市场。完成汇丰、清源、金诚三家公司1334万吨原油进口资质的审批工作，组织好原油直接进口；积极扩大先进技术、重大装备、关键零部件进口，加快企业装备升级。制定全市“一带一路”产业合作指导目录，推进柬埔寨齐鲁工业园等境外经济园区建设，鼓励更多有比较优势的企业“走出去”。

三是持续打造一流营商环境。对“三最城市”指标体系实行动态管理，始终保持审批事项最少、收费标准最低、审批时限最短。健全完善网上审批大厅，推进建设项目前置审批区域化评估评审。严格执行行政权力清单和政府责任清单，坚决杜绝清单之外的行政执法，严肃查处各类“乱作为”。加快市电子政务和信息中心建设，实施云计算、大数据等信息资源的整合。大力加强社会信用体系建设，加快完善企业信息公示和“黑名单”制度，重点推进政务诚信、商务诚信、社会诚信和司法公信，努力建设“信用淄博”。

四、进一步加强政府自身建设

打铁还需自身硬。面对“十三五”时期提前全面建成小康社会的宏伟目标和艰巨繁重的改革发展任务，必须加快转变政府职能，全面加强政府自身建设，推进政府治理体系和治理能力现代化。

一是在法治政府建设上实现新突破。健全完善法治政府建设推进机制，严格按照法定权限和法定程序行使权力、履行职责，坚决做到法定职责必须为、法无授权不可为。完善重大行政决策专家咨询和法律顾问制度，确保决策制度科学、程序正当、过程公开。全面落实行政执法责任制，严格规范公正文明执法，更好地运用法治思维和法治手段化解矛盾、推动工作。自觉执行市人大及其常委会的决议决定，积极支持人民政协履行职能，认真听取民主党派、工商联、无党派人士、人民团体意见，主动接受人大、政协和社会各界监督，让权力在阳光下运行。

二是在效能政府建设上实现新突破。深化行政审批制度改革，大力推进网上审批、联审代办和建设项目“一费制”，为市场主体提供高效优质服务。建立第三方评估机制和责任倒查机制，确保严格兑现服务承诺。坚持把狠抓落实作为政府工作的生命线，强化问题导向和目标管理，对重点工作、重点任务，立下军令状、制定路线图、列出时间表，做到责任倒逼、时间倒推、工期倒排，以踏石留印、抓铁有痕的劲头推动工作落实。持之以恒加强作风建设，坚决纠正不作为、慢作为，坚决克服懒政、怠政，坚决查处失职、渎职，坚决做到有错必纠、有责必问。

三是在廉洁政府建设上实现新突破。严格执行中央和省市委加强党风廉政建设的各项要求，把守纪律讲规矩摆在更加突出的位置，认真履行政府党风廉政建设主体责任，深入开展廉政教育，切实加强重点部门和关键岗位廉政风险防控，坚持用制度管权管人管事。强化行政监察，充分发挥审计监督作用，加大对公共资金、公共资源、国有资产的常态化监管，严格控制“三公”经费，把有限的资源和财力用在推动发展、改善民生上。

各位代表！蓝图催人奋进，拼搏铸就辉煌。让我们紧密团结在以习近平同志为总书记的党中央周围，在中共淄博市委的坚强领导下，全面贯彻党的十八大和十八届三中、四中、五中全会精神，解放思想、奋力开拓，为提前全面建成小康社会、开创淄博更加美好的未来而努力奋斗！
